# Racial disparities and maternal mortality in Brazil: findings from a national database

**DOI:** 10.11606/s1518-8787.2024058005862

**Published:** 2024-06-20

**Authors:** Amanda Dantas Silva, José Paulo Siqueira Guida, Debora de Souza Santos, Silvia Maria Santiago, Fernanda Garanhani Surita

**Affiliations:** I Universidade Estadual de Campinas Faculdade de Ciências Médicas Departamento de Tocoginecologia Campinas SP Brasil Universidade Estadual de Campinas. Faculdade de Ciências Médicas. Departamento de Tocoginecologia. Campinas, SP, Brasil; II Universidade Estadual de Campinas Faculdade de Enfermagem Campinas SP Brasil Universidade Estadual de Campinas. Faculdade de Enfermagem. Campinas, SP, Brasil; III Universidade Estadual de Campinas Faculdade de Ciências Médicas Departamento de Saúde Coletiva Campinas SP Brasil Universidade Estadual de Campinas. Faculdade de Ciências Médicas. Departamento de Saúde Coletiva. Campinas, SP, Brasil

**Keywords:** Health Disparities, Racism, Women’s Health Services

## Abstract

**OBJECTIVE:**

To assess maternal mortality (MM) in Brazilian Black, Pardo, and White women.

**METHODS:**

We evaluated the maternal mortality rate (MMR) using data from the Brazilian Ministry of Health public databases from 2017 to 2022. We compared MMR among Black, Pardo, and White women according to the region of the country, age, and cause. For statistical analysis, the Q^2^ test prevalence ratio (PR) and confidence interval (CI) were calculated.

**RESULTS:**

From 2017 to 2022, the general MMR was 68.0/100,000 live births (LB). The MMR was almost twice as high among Black women compared to White (125.81 vs 64.15, PR = 1.96, 95%CI:1.84–2.08) and Pardo women (125.8 vs 64.0, PR = 1.96, 95%CI: 1.85–2.09). MMR was higher among Black women in all geographical regions, and the Southeast region reached the highest difference among Black and White women (115.5 *versus* 60.8, PR = 2.48, 95%CI: 2.03–3.03). During the covid-19 pandemic, MMR increased in all groups of women (Black 144.1, Pardo 74.8 and White 80.5/100.000 LB), and the differences between Black and White (PR = 1.79, 95%CI: 1.64–1.95) and Black and Pardo (PR = 1.92, 95%CI: 1.77–2.09) remained. MMR was significantly higher among Black women than among White or Pardo women in all age ranges and for all causes.

**CONCLUSION:**

Black women presented higher MMR in all years, in all geographic regions, age groups, and causes. In Brazil, Black skin color is a key MM determinant. Reducing MM requires reducing racial disparities.

## INTRODUCTION

Reducing maternal mortality is a global health concern included in the United Nations Sustainable Development Goals (SDG). The SDG recognizes that ending poverty and other deprivations involves health improvement and reducing inequalities by setting 17 specific goals. Goal number 3 (SDG3) refers to health and well-being for all, SDG5 strives for gender equity, and SDG10 focuses on reducing inequities^1^. Reducing maternal mortality, increasing the quality of maternal care, and ensuring universal access to sexual and reproductive health services are among the goals. In this sense, improving health measures, promoting gender equity, and reducing inequities are vital to reducing maternal mortality^2^.

Social conditions of health determine health inequities. These social determinants are non-medical conditions associated with where people live, work, and grow, which impact their risk factors and health outcomes^3^. Structural determinants include socioeconomic and political contexts that create and maintain social hierarchies through which populations are stratified according to gender, race, skin color, income, and education^4^. These structural factors contribute to the generation of differential health risks. In this context, structural racism is a population health determinant that negatively impacts health outcomes^5,6^. Racism is a system of domination of a racial group defined as inferior by dominant groups that use phenotypic characteristics to justify inequalities in access to resources and power. In Brazil, the characteristics and duration of slavery, the way in which abolition occurred, the arrival of European immigrants, miscegenation, and the myth of racial democracy contributed to the perpetuation of structural racism. Racism permeates pregnancy outcomes: Black women are at a higher risk of maternal and perinatal adverse outcomes and report worse experiences during prenatal, childbirth, and postpartum care^7^.

Black women suffer from gender, social, and racial intersecting vulnerabilities with multiplicative effects produced by a historical context of discrimination and oppression that leads to unequal access to resources, representation, and health facilities^10,11^.

Maternal mortality reflects inequalities more than any other measure of health^12^. Maternal mortality is higher in low-income countries and is impacted by poor access to health education and higher gender inequality. It is also affected by race, ethnicity, and external factors such as humanitarian crises^2^.

Although global maternal mortality has reduced in the last 20 years, this progress is uneven: significant inequalities persist between regions and countries. Most maternal deaths occur in low- and lower-middle-income countries and are preventable. The death of a woman due to avoidable causes is an indicator of her status in society and of the inadequacy of the healthcare system to meet the needs of women as a social group. It highlights the disparities pervading health issues^2,13^.

Maternal mortality in Brazil has substantially decreased since 1990. From 1990 to 2010, the maternal mortality rate (MMR) showed a sharper drop, followed by a stabilization trend until 2019. The pandemic period was an outlier in maternal mortality rates, with an expressive rise from 2020 to 2022. Few studies have analyzed the trend in maternal mortality according to skin color in Brazil, despite growing evidence of racial disparities in health. The COVID-19 pandemic was a public health emergency that rapidly increased maternal deaths worldwide; in Brazil, maternal mortality excess due to the pandemic was 1.40-fold in Brazil^2^.

This study aims to assess maternal mortality (MM) in Brazil according to skin color and compare MM in Black, Pardo, and White women, considering geographical determinants, age group, pre-pandemic and pandemic periods, and causes of mortality.

## METHODS

We conducted a retrospective cross-sectional study to assess the relationship between skin color and maternal deaths in Brazil. According to the World Health Organization (WHO), maternal mortality is the annual number of female deaths from any cause related to or aggravated by pregnancy or its management (excluding accidental or incidental causes) during pregnancy and childbirth or within 42 days of termination of pregnancy, irrespective of the duration and site of the pregnancy.

We analyzed two public databases maintained by the Brazilian Ministry of Health. The first database was the Brazilian Ministry of Health Panel of Living Births (LB), and the second was the Brazilian Ministry of Health Panel of Maternal Mortality (MM). According to local regulations, all LB and maternal deaths (MD) must be reported to the authorities, and these data are anonymized and made public in the panels mentioned above. We evaluated data on Maternal Mortality Rate (MMR) from 2017 to 2022 for each year and the entire period. MMR is defined as the division of MD by LB, expressed in every 100,000 LB.

The two above-mentioned Panels contains data that werecollected from two mandatory reporting instruments in Brazil: the Live Birth Certificate and the Death Certificate. These forms are provided to all healthcare services by local health departments, and their information are verified by respective state health departments, with the Ministry of Health compiling and anonymizing all data. We collected data on skin color, maternal age, geographic area, and cause of maternal death from these databases. Access to both Panels is public through the Internet.

The Panels mentioned above and the Brazilian Institute of Geography and Statistics (IBGE) classify the Brazilian population based on skin color self-declaration as White, Black, Pardo, Yellow (East Asian), Indigenous, or Ignored. In Brazil, ethnicity is particularly complex due to great miscegenation, and we choose to use the word Pardo (instead of Brown or Mixed Brown, as in other studies), as it comes from the self-declaration of the women and is used in Brazilian Portuguese. In this study, we initially presented the MMR for all categories. For bivariate analysis, we compared women with self-declared skin colors as Black, Pardo, and White, comparing White women versus Black women and Pardo women versus Black women. We decided to exclude Indigenous and Yellow women from the comparative analysis for two reasons: first, other factors are related to inequities among Indigenous peoples, and second, the low prevalence of Yellow women in the general population (1.16%)^14^.

We compared MMR among self-referred women of Black, Pardo, and White skin color according to regions of Brazil (North, Northeast, South, Southeast, and Midwest), maternal age (0–19, 20–29, 30–39, 40 or more), cause of MM (direct causes, including hypertension, hemorrhage, infection, and abortion; and indirect causes, those that cannot be categorized among the direct causes), and in periods prior (2017–2019) and during the Coronavirus pandemic (2020–2022). For statistical analysis, the Chi-squared test and prevalence ratio (PR) and confidence interval (CI) were obtained using EpiInfo 7.0. A significance level of 5% was adopted.

We decided to adopt the group of White and Pardo women as the reference to compare maternal deaths among Black women because we understand that Black women suffer from the effects of Racism in their health outcomes in comparison to the other two groups.

The analysis of this study used a public national database available online and therefore does not require a consent form, as per the Declaration of Helsinki as amended in Hong Kong in 1964.

According to Resolution 510/2016 of Ethics in Research, the following will not be registered or evaluated by the system of the National Committee for Ethics in Research in Brazil: “Research that uses publicly accessible information, under the terms of Law No. 12,527, of November 18, 2011; research that uses information in the public domain”.

## RESULTS

From 2017 to 2022, there were 10,743 cases of MD and 158,06,414 cases of LB in Brazil. The general MMR in the study period was 68.0/100,000 LB and 125.8/100,000 LB, 64.0/100,000 LB, and 64.1/100,000 LB for Black, Pardo, and White women, respectively. Black women presented a higher MMR in all years of the considered period. The year 2021 gave the highest general MMR (110.8), with an MMR of 190.8 among Black women. The total MMR in the pre-pandemic period (2017 to 2019) was 56.8, whereas during the pandemic period (2020 to 2022) rose to 81.7.

**Figure 1:**
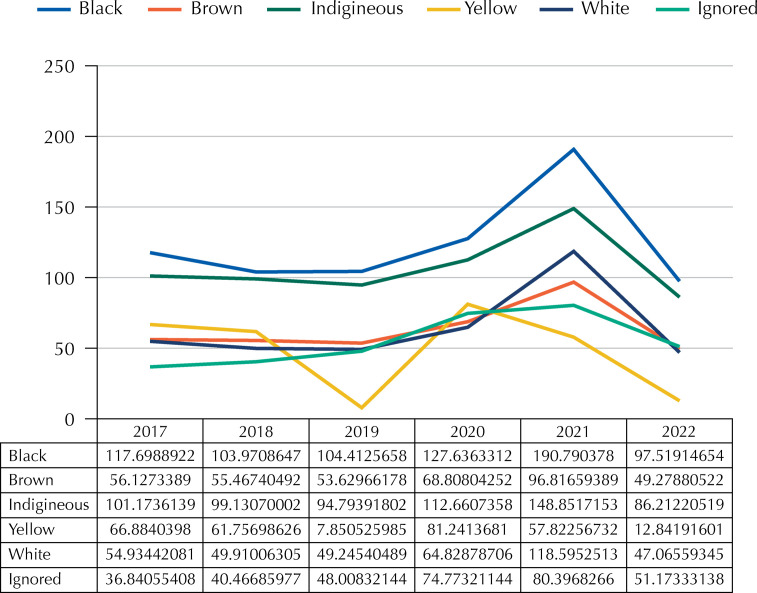
Maternal Mortality Rate in Brazil between 2017 to 2022, according to skin color, expressed per 100.000 live births.

Figure 1 is a graphic presentation of MMR according to skin color in all categories.

The MMR was almost twice as high among Black women compared with White and Pardo women over the entire evaluated period. In all years, MMR was significantly higher among Black women in comparison to White or Pardo women. Black women also presented higher MMR than White and Pardo women during the pandemic.

The MMR among White, Black, and Pardo women through the years and comparisons between the pre-pandemic and pandemic periods are shown in Table 1.

**Table 1:** MMR according to skin color (Black, Pardo, and White) by year (2017 to 2022) and period (pre and during the coronavirus pandemic).

**Skin color**	**Black**	**White**	**Pardo**	**Black versus White**		**Black versus Pardo**	
	**MMR**	**MMR**	**MMR**	**PR (95%IC)**	**p-value**	**PR (95%IC)**	**p-value**
Total	125.8	64.1	64.0	1.96 (1.84–2.08)	< 0.001	1.96 (1.85–2.09)	< 0.001
Year							
2017	117.7	54.9	56.1	2.14 (1.81–2.53)	< 0.001	2.10 (1.79–2.45)	< 0.001
2018	104.0	49.9	55.5	2.08 (1.75–2.47)	< 0.001	1.87 (1.59–2.20)	< 0.001
2019	104.4	49.2	53.6	2.12 (1.79–2.51)	< 0.001	1.95 (1.66–2.28)	< 0.001
2020	127.6	64.8	68.8	1.97 (1.69–2.29)	< 0.001	1.85 (1.61–2.14)	< 0.001
2021	190.8	118.6	96.8	1.61 (1.42–1.82)	< 0.001	1.97 (1.75–2.21)	< 0.001
2022	97.5	47.1	49.3	2.08 (1.67–2.58)	< 0.001	1.98 (1.61–2.42)	< 0.001
Period							
Pre-pandemic (2017–2019)	108.4	51.4	55.1	2.11 (1.91–2.32)	< 0.001	1.97 (1.79–2.16)	< 0.001
COVID-19 Pandemic (2020–2021)	144.1	80.5	74.8	1.79 (1.64–1.95)	< 0.001	1.92 (1.77–2.09)	< 0.001

**Figure 2:**
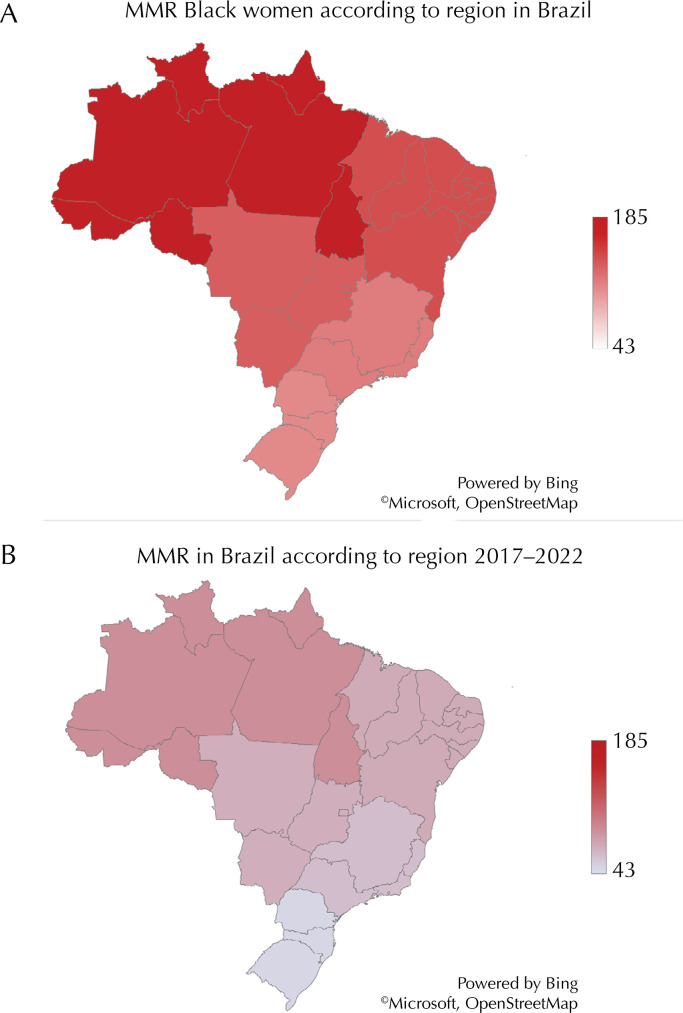
Geographical distribution of MMR in Brazil, according to skin color. Figure 2A shows MMR among Black women. Figure 2B shows MMR among all women using the same scale.

In all geographical regions of Brazil, MMR was higher among Black women, reaching 186/100,000 LB in the North region. In the Southeast region (the richest of the country), the difference in MD between Black and White women was the largest (115.5 *versus* 60.8, PR 2.48 CI 2.03 – 3.03, p< 0.001), and only in the North region, there was no difference in the prevalence of MD among both groups. When comparing Black to Pardo women, MD was significantly higher among Black women in all country regions. Figure 2 shows the geographical distribution of MMR in Brazil according to skin color.

MMR increased with age in all three considered groups; however, in all age ranges, it was significantly higher among Black women than among White or Pardo women. The largest difference in MMR between Black and White women was observed in the 30–39 age group (167.5 *versus* 74.9, PR = 2.24, 95%CI: 2.03–2.46, p < 0.001), and between Black and Pardo women was observed in the 20-29 age group (97.1 *versus* 50.3, PR = 2.01, 95%CI: 1.82–2.22, p < 0.001).

Concerning the cause of maternal mortality, Brazilian women died primarily because of direct causes, and hypertension was the leading cause of maternal death in the study period. The MMR due to indirect causes was 29.9, and that from direct causes was 37.7 during the period. Black women had a higher MMR for all the causes than white women, as shown in Table 2.

**Table 2:** MMR according to skin color (Black, Pardo, and white) by age group, region, and cause.

**Variables**	**Black**	**White**	**Pardo**	**Black versus White**		**Black versus Pardo**	
	**MMR**	**MMR**	**MMR**	**PR (95%IC)**	**p-value**	**PR (95%IC)**	**p-value**
Age							
0–19	82.8	49.0	42.4	1.69 (1.35–2.11)	< 0.001	1.95 (1.60–2.39)	< 0.001
20–29	97.1	50.3	48.3	1.93 (1.74–2.15)	< 0.001	2.01 (1.82–2.22)	< 0.001
30–39	167.5	74.9	93.3	2.24 (2.03–2.46)	< 0.001	1.79 (1.63–1.97)	< 0.001
40 or more	301.0	141.3	178.3	2.13 (1.71–2.65)	< 0.001	1.69 (1.37–2.08)	< 0.001
Region							
North	186.0	182.2	75.9	1.02 (0.79–1.31)	0.924	2.45 (1.96–3.06)	< 0.001
Northeast	141.8	106.6	66.1	1.33 (1.16–1.52)	< 0.001	2.14 (1.92–2.40)	< 0.001
South	108.0	43.6	56.7	1.90 (1.73–2.08)	< 0.001	2.07 (1.89–2.28)	< 0.001
Southeast	115.5	60.8	55.7	2.48 (2.03–3.03)	< 0.001	1.90 (1.51–2.41)	< 0.001
Midwest	132.7	83.9	65.7	1.58 (1.24–2.00)	< 0.001	2.02 (1.61–2.53)	< 0.001
Cause							
Indirect	51.2	28.9	25.1	1.77 (1.60–1.96)	< 0.001	2.04 (1.85–2.25)	< 0.001
Direct (all)	70.8	32.9	36.9	2.15 (1.97–2.35)	< 0.001	1.92 (1.77–2.08)	< 0.001
Hypertension	25.6	8.97	10.9	2.85 (2.45–3.32)	< 0.001	2.35 (2.05–2.70)	< 0.001
Postpartum hemorrhage	10.1	6.70	7.10	1.52 (1.21–1.89)	< 0.001	1.43 (1.16–1.76)	< 0.001
Miscarriage	3.14	1.80	1.90	1.75 (1.17–6.22)	0.009	1.65 (1.12–2.42)	0.013
Infection	5.48	2.02	2.95	2.71 (1.95–3.75)	< 0.001	1.86 (1.38–2.49)	< 0.001

## DISCUSSION

Our results showed that MMR in Brazil was significantly higher among Black women than among White or Pardo women in all years available, before and during the COVID-19 pandemic, in all Brazilian geographical regions and age groups. We believe that our results demonstrate the impact of racism on maternal mortality. The increased rates of deaths among Black women are a consequence of a social construction that negatively impacts their health outcomes and are not related to any genetic or biological factors.

The maternal mortality ratio among Black women was consistently higher, regardless of the pandemic public health emergency. Previous studies have shown racial disparities in maternal mortality during the covid-19 pandemic^15,16^, but our findings highlight the preexisting inequities and the underlying structural inequalities and reinforce that access to health care has been impaired, as other studies have already demonstrated. However, this access was probably more impaired for Black women, which impacted maternal mortality. A cross-sectional study using maternal mortality rates in the USA from 2000 to 2019^17^, as well as Brazilian national data from the same period^18^, observed higher MMR among Black women, which demonstrates the need to examine the pre-pandemic status of vulnerable populations^19^.

In our analysis, MMR was higher among Black women in all geographical regions of Brazil. Compared with Pardo women, Black women had higher MMR in all country regions. Our country’s regional differences in maternal mortality rates are well known^20,21^, with higher MMR among low-income regions^18^. However, our study showed racial disparities in all regions, even those with higher resource availability. Socioeconomic and resource availability differences are often used to justify racial differences in health outcomes, but our findings illustrate how implicit bias — attitudes that unconsciously affect our understanding and decisions — can contribute to health inequalities^11^.

MMR was significantly higher among Black women than among White or Pardo women in all age groups. Higher MMR rates are expected in extreme age groups. A systematic analysis using mortality estimates from the Global Burden of Disease Study database that evaluated the maternal mortality ratio in Brazil from 1990 to 2019^18^ showed that in Brazil, MMR is highest in extreme age groups and lowest in the 20–24 age group. Another systematic review of global data also concluded that advanced maternal age is a risk factor for maternal mortality^23^. Our analysis, otherwise, demonstrated a higher MMR among Black women, compared to White and Pardo women, not only in the extreme age groups but also in the 20–39 age group, when maternal death is even less expected. In our study, women died primarily because of direct causes, and hypertension was the leading cause of maternal death. These findings align with previous literature: a WHO systematic analysis of global data showed that most maternal deaths were due to direct causes, and hemorrhage was the leading cause^2^; however, in Brazil, hypertension seems to consistently be the leading cause of maternal deaths^18^. It is already known that Black women are disproportionally affected by hypertensive disorders during pregnancy^25^, and our results confirmed this inequality. Many theories explain these racial differences in hypertension during pregnancy, but the underlying rationale for it is multifactorial^26^. Some studies have proposed that underlying comorbidities are more prevalent among Black women and other socioeconomic factors, such as lack of access to quality care, education, and lower income, to explain the racial disparities. Even after adjusting for these variables, Black skin color was independently associated with an increased risk of hypertension in pregnancy^25^.

Strategies have been proposed to reduce maternal mortality. The main proposals are to improve access to health services and the quality of care from preconception to postpartum through prenatal care^27,28^. Considering that Black women have a higher risk of inadequate prenatal care and lack of attachment to motherhood^29^, reducing racial disparities in obstetric care is crucial for reducing maternal mortality.

Addressing health disparities requires a multidomain approach beyond the traditional link between illness and medical interventions. To reduce racial differences in maternal mortality, we need to consider structural racism, intersectional vulnerabilities, and social inequities^30,31^

The National Institute on Minority Health and Health Disparities (NIMHD) has developed a research framework tool “to understanding and addressing minority health and health disparities and promoting health equity.” This framework pleads the need for attention to biological and social determinants of health rather than an exclusive focus on one or the other^32^. Researchers must consider the variable skin color within a historical context of discrimination as a complex variable that interferes with health outcomes not only because of genetic and biological factors^33^. Acknowledging racism and the existence of racial disparities in decision making and the construction of public policies makes it possible to reduce health inequalities^34^.

The causal pathways between racism and maternal health inequalities are complex and multifaceted, reflecting the pervasive impact of systemic racism on various aspects of healthcare. Discrimination and bias in healthcare settings can lead to disparities in access to quality prenatal care and maternal services for racial and ethnic minority groups. Additionally, socioeconomic factors influenced by systemic racism, such as income inequality and neighborhood segregation, contribute to disparities in maternal health outcomes. Chronic stress resulting from experiences of racism can also negatively affect maternal health, potentially leading to preterm births and low birth weights. Furthermore, inadequate representation of minority groups in healthcare decision-making and policy formulation can perpetuate disparities by failing to address the specific needs of these populations. To effectively address maternal health inequalities, it is crucial to recognize and dismantle the structural and institutional barriers rooted in racism that impact maternal care and outcomes.

Our study has some limitations. We obtained data from national databases with already stratified data, limiting multivariate analysis, for example. In addition, maternal deaths in Brazil are underreported. In Brazil, the classification and identification of race/ethnicity is complex due to multiple factors: historical oppression built based on a slave-owning society, racial democracy myth, miscegenation as a “population whiten” tool, and unconscious racism affect rates of self-declaration as Black. The discriminated population itself does not recognize themselves as discriminated against because they do not want to identify themselves as Black.

One of the strengths of our analyses is the large sample size, which provides sufficient statistical power for the associations. In addition, this was a population-based study, allowing reproduction of the results.

In summary, the MMR among Brazilian Black women is higher than that among White or Pardo women. MMR is consistently higher among black women in all Brazilian geographic regions, age groups, and causes. In Brazil, Black skin color plays a relevant role as a factor in determining MM. It is essential to recognize it in all levels of care: during antenatal care, delivery, and postpartum period, by early detection of maternal morbidity and treatment of potentially life-threatening conditions. It is also necessary that health professionals reflect on the impact of Racism in their daily activities in care, and health managers provide adequate training to avoid Racism in health care and also punish those events. Finally, it is mandatory to consider maternal mortality as a priority in research and to include the racial perspective in these studies.
